# A projection-based inverse kinematic model for extensible continuum robots and hyper-redundant robots with an elbow joint

**DOI:** 10.3389/frobt.2025.1627688

**Published:** 2025-09-12

**Authors:** Sven Fritsch, Dirk Oberschmidt

**Affiliations:** Department of Mechanical Engineering and Transport Systems, Technical University, Berlin, Germany

**Keywords:** continuum robots, hyper-redundant robots, inverse kinematics, optimization algorithm, robot control

## Abstract

Inverse kinematics is a core problem in robotics, involving the use of kinematic equations to calculate the joint configurations required to achieve a target pose. This study introduces a novel inverse kinematic model (IKM) for extensible (i.e., length-adjustable) continuum robots (CRs) and hyper-redundant robots (HRRs) featuring an elbow joint. This IKM numerically solves a set of equations representing geometric constraints (abbreviated as NSGC). NSGC can handle target poses $X_{t}=\left[x_{t},y_{t},z_{t},\psi_{t}\right]$ in 3D space, which are projected onto a 2D plane and solved numerically. NSGC is capable of real-time operation and accounts for elbow joint limits. Extensive simulations and empirical tests confirm the reliability, performance, and practical applicability of NSGC.

## 1 Introduction

In recent years, the design and modeling of continuum robots (CRs) and hyper-redundant robots (HRRs) have garnered significant attention. CRs comprise multiple backbones routed in parallel and attached to a common end disk. Typically, CRs also incorporate spacer disks to maintain the backbone configuration and prevent buckling during actuation. On the other hand, HRRs consist of multiple rigid elements that can rotate relative to one another, enabling highly flexible and dexterous movements. Bending of the HRR is achieved through tendons routed through each segment. These tendons are connected to actuators that apply tension, inducing the desired curvature. Additional tendons control instruments located at the HRR’s end-effector. The inherent miniaturization potential of CRs and HRRs makes them well-suited for applications in minimally invasive surgery and endoscopy, where precision and maneuverability are essential.

### 1.1 A literature review of IKMs for CRs and HRRs

The most commonly used approach to modeling CRs and HRRs is the constant curvature model. CRs and HRRs with multiple segments can be modeled using the piecewise constant curvature model (Burgner-Kahrs et al., 2015). Using the Jacobian matrix to establish differential kinematics is the standard approach for CR/HRR inverse kinematic models (IKMs) (Whitney, 1972) and was also employed by Wolf et al. (2005), Garg et al. (2014), Guardiani et al. (2022), Lee et al. (2014), Li et al. (2017), Rone and Ben-Tzvi (2014), Simaan et al. (2009), Jones and Walker (2006), and Yeshmukhametov et al. (2019).

The generalized coordinates q are updated by the multiplication of the pseudo-inverse of the Jacobian (Chirikjian and Burdick, 1994) by the difference in parameterized end-effector coordinates (Klein and Huang, 1983). Alternative approaches comprise the augmented (Seraji, 1989) and extended (John, 1983) Jacobian.

Bajo et al. (2012) applied a Jacobian-based IKM to their IREP platform, which consists of two independent, two-segment CRs. They computed the Jacobian matrices for each segment individually.

Giorelli et al. (2013) proposed an alternative approach tailored to non-constant curvature CRs, where the static model is governed by nonlinear differential equations, making exact solutions challenging. As analytical solutions are unavailable, the elements of the Jacobian matrix cannot be computed directly. Instead, a feed-forward neural network is trained to learn the IKM for a non-constant curvature cable-driven manipulator. Similar neural network-based methods were presented by Lai et al. (2019) and Thuruthel et al. (2016).

Wei et al. (2023) presented an IKM for a two-segment HRR under the constant curvature assumption. They simplified the complex nonlinear system by reducing it to a single nonlinear equation, resulting in faster solutions.

Xu et al. (2018) adopted an iterative approach to compute the IKM utilizing a least squares method to determine the joint angles from specified cable lengths. Their model incorporated multilevel mappings that describe the relationships between motors and cables, cables and joints, and joints and the robot’s end-effector.

Almanzor et al. (2023) introduced a method that combines a local inverse kinematics formulation in image space with a deep convolutional neural network trained on synthetic data. A 2D hand-drawn image of the desired shape is used as the input, and the network predicts motor commands to drive the robot toward that shape. Closed-loop control is achieved using visual feedback from a webcam.

Wild et al. (2023) developed a novel piecewise dual quaternion algorithm to model multi-section CRs, aiming to improve the traditional pseudo-inverse Jacobian method. The piecewise dual quaternion approach offers reduced computational complexity, faster convergence, and smoother backbone configurations, especially as the number of robot sections increases.

Kim and Wi (2025) introduced a tendon-driven discrete CR using ball–socket joints. Between one and three robot units connected in series were tested by applying proximal tendon tension, while distal tension was gradually increased to induce bending. The resulting bending curves were interpolated using third-to-fifth-order polynomials.

Li et al. (2025) proposed a novel multilevel motion control method for HRRs based on joint angle–cable force cooperative optimization. It comprises a finite-time optimization approach using macro–micro decomposition to solve inverse kinematics. The high-degree-of-freedom (DoF) robot is divided into smaller manipulators and solved using a dual neural network model.

Using the space vector method, the manipulator’s kinematic model is developed to dynamically determine its endpoint position (Wang et al., 2024). The workspace is then generated through the Monte Carlo method. The original search approach is enhanced by introducing an angle-decoupling mechanism between adjacent links to calculate each joint’s rotation angles.

Zhan et al. (2024) introduced a general approach for solving the real-time optimized IKM of redundant robots, while strictly enforcing hard limits in both joint and Cartesian spaces that cannot be violated. Instead of quadratic programming, the method employs constrained linear programming to address the IKM problem. Hard constraints—including joint limits, velocity, and acceleration bounds—are explicitly managed as inequality constraints.

Sheng et al. (2024) presented an IKM method that combines the end-following approach with a segmented, Jacobian-based iterative solver for CRs with redundant DoF and a moving base. The end-following method is first used to generate a smooth, constraint-compliant initial joint configuration by having joints sequentially follow the target points along a planned path. This initial guess is then refined using Jacobian-based inverse kinematics applied to robot segments, thus improving accuracy while reducing the computation time.

The following subsection provides a brief overview of the Jacobian-based IKM.

### 1.2 Jacobian-based IKM

The state-of-the-art IKMs for CRs and HRRs are based on the Jacobian matrix J . q denotes the generalized (joint) coordinates, and X(q) denotes the end-effector pose corresponding to q . ${\left(q\right)}_{0}$ represents the initial guess of q . $X_{t}$ denotes the target pose. $J^{+}$ represents the Moore–Penrose inverse of the Jacobian as shown in Algorithm 1.

Algorithm 1 Jacobian-based IKM.

1: Initialize tol←0.01 and $q\leftarrow {\left(q\right)}_{0}$

2: **while** $\|X_{t}-X\left(q\right)\|>tol$ **do**
3:  **Compute** J(q)

4: ${J\left(q\right)}^{+}\leftarrow {\left(J\left(q\right)\right)}^{+}$

5: $\Delta X\leftarrow X_{t}-X\left(q\right)$

6: $q\leftarrow q+k\cdot {J\left(q\right)}^{+}\cdot \Delta X$

7: **end while**

It should be noted that, in general, calculating the orientation difference requires careful handling beyond the simple subtraction of the target and achieved end-effector orientations.

The Jacobian matrix, as defined in Equation 1, is commonly used in the kinematics and dynamics of robotic systems. It relates variations in joint space to corresponding changes in configuration space.

$$J\left(q\right)=\left[\begin{array}{ccc} \frac{\partial X_{1}}{\partial q_{1}} & \cdots & \frac{\partial X_{1}}{\partial q_{n_{j}}}\\ \vdots & \ddots & \vdots \\ \frac{\partial X_{m}}{\partial q_{1}} & \cdots & \frac{\partial X_{m}}{\partial q_{n_{j}}} \end{array}\right].$$ (1)

The forward kinematic model of the robot describes the transformation matrices from the inertial frame I to the end-effector frame E , as provided in Equation 2.

$$T_{\mathrm{I E}}\left(q\right)=T_{I0}\cdot \left(\overset{m}{\underset{k=1}{\prod }}T_{k-1,k}\left(q_{k}\right)\right)\cdot T_{mE}.$$ (2)

The Moore–Penrose inverse is computed using Equation 3.

$$J^{+}={\left(J^{T}J\right)}^{-1}J^{T}.$$ (3)

Due to the iterative nature of the algorithm, the error converges against a predefined threshold that is considered acceptable by the user. The gain k increases the step size per update and influences the convergence speed.

This study introduces a novel IKM that departs from the traditional Jacobian-based approach. Instead, it relies on the numerical solution of a set of equations representing geometric constraints, referred to hereafter as NSGC. These equations are formulated using the unknown parameters of the arc representing the curvature of the bent CR/HRR. Solving this system enables the calculation of the robot’s length and bending parameters, which are subsequently converted into generalized coordinates q .

## 2 NSGC

**FIGURE 1:**
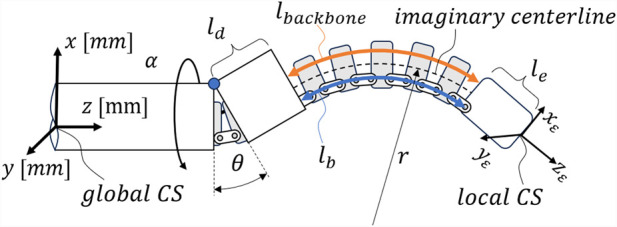
General CR/HRR structure suitable for NSGC: a proximal part that can be rotated about its main axis about angle α is followed by an elbow joint with deflection angle θ , a distal part with length $l_{d}$ , a length-adjustable bending part characterized by length $l_{b}$ and $\Delta l_{b}$ , and the end-effector with length $l_{e}$ ; CS stands for coordinate system.

Given a target pose $X_{t}=\left[x_{t},y_{t},z_{t},\psi_{t}\right]$ in 3D space, the objective of the NSGC algorithm is to compute the generalized coordinates q defined by $q=\left[l_{b},\Delta l_{b},\alpha ,\theta \right]$ . For CRs and HRRs, $l_{b}$ is defined as the length of a tendon/backbone, while $\Delta l_{b}$ is the length difference of the antagonistically actuated tendon/backbone. As the proposed HRR only features one backbone, $l_{b}$ defines the sum of the lengths of the robotic elements and the connectors between the distal end of the distal part and the proximal end of the end-effector, while $\Delta l_{b}=l_{b}-l_{\mathrm{backbone}}$ defines the length difference between the backbone and $l_{b}$ , as illustrated in Figure 1. The proposed NSGC method can be applied to CRs and HRRs with an elbow joint and adjustable length capabilities.

### 2.1 Projection from 3D space onto the 2D plane

The target pose $X_{t}=\left[x_{t},y_{t},z_{t},\psi_{t}\right]$ is given in 3D space. $x_{t},y_{t}$ and $z_{t}$ are the position coordinates defined in the global CS, while $\psi_{t}$ describes the end-effector orientation about the $y_{\varepsilon }$ -axis expressed in the local end-effector CS. The target pose must be projected onto a 2D plane, i.e., the global x–z-plane with $\text{proj}\left(X_{t}\right)={\hat{X}}_{t}=\left[{\hat{x}}_{t},z_{t},\psi_{t}\right]$ . The projection ${\hat{x}}_{t}$ is computed using Equation 4 and can be either positive or negative.

$${\hat{x}}_{t}=\pm \sqrt{x_{t}^{2}+y_{t}^{2}}.$$ (4)

**FIGURE 2:**
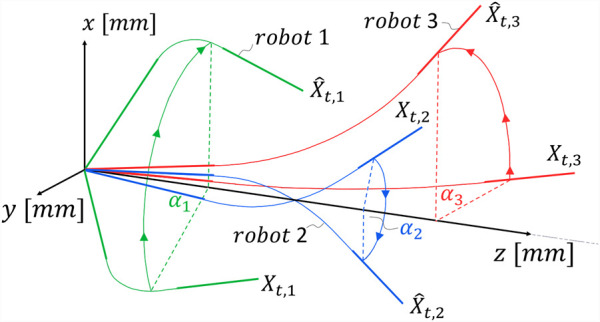
Projection of target poses $X_{t}$ defined in 3D space projected onto the global x–z-plane.

As shown in Figure 2, the angle α , about which $X_{t}$ is rotated, is defined by Equation 5.

$$\alpha =\text{atan}2\left(y_{t},x_{t}\right).$$ (5)

The projection does not affect $\psi_{t}$ because $\psi_{t}$ is defined as the rotation about the $y_{\varepsilon }$ -axis in the local end-effector frame. In other words, $\psi_{t}$ represents the same local orientation in both the original and projected poses. Similarly, the z-axis also remains unchanged by the projection, i.e., the z-value of the original target pose $X_{t}$ in 3D is identical to the z-value of the projected target pose ${\hat{X}}_{t}$ in 2D.

The projection of different robot configurations onto the global x–z-plane is displayed in Figure 2. $X_{t}$ denotes the original target pose, as defined by the user, and ${\hat{X}}_{t}$ denotes the target pose projected onto the global x–z-plane. The angle of rotation of the pose is denoted by α . For example, the $X_{t,1}$ pose is rotated about $\alpha_{1}=-60{}^{\circ}$ ; the $X_{t,2}$ pose is rotated about $\alpha_{2}=-155{}^{\circ}$ ; and the $X_{t,3}$ pose is rotated about $\alpha_{3}=90{}^{\circ}$ . *A priori*, it is unknown whether the projection ${\hat{x}}_{t}$ should be positive (e.g., $X_{t,1}$ or $X_{t,3}$ ) or negative $\left(X_{t,2}\right)$ . A detailed treatment of the projection ambiguity is provided in Section 2.1.

### 2.2 Robot configurations in 2D

**FIGURE 3:**
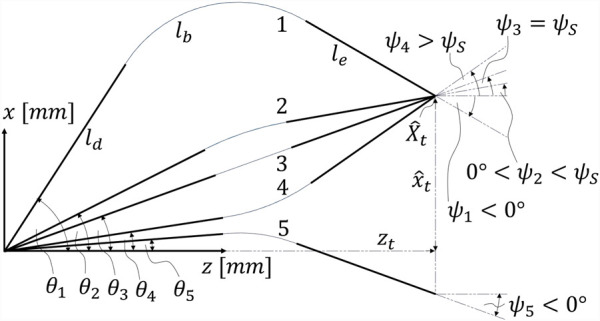
Different cases: robot 1: downward bending with $\psi_{1}<$ 0° and ${\hat{x}}_{t}>0$ ; robot 2: downward bending with 0° $<\psi_{2}<\psi_{S}$ and ${\hat{x}}_{t}>0$ ; robot 3: straight configuration with $\psi_{3}=\psi_{S}$ and ${\hat{x}}_{t}>0$ ; robot 4: upward bending with $\psi_{4}>\psi_{S}$ and ${\hat{x}}_{t}>0$ ; robot 5: downward bending with $\psi_{5}<$ 0° and ${\hat{x}}_{t}<0$.

Given a CR or HRR with an elbow joint and a length-adjustable bending part, the robot can achieve target poses ${\hat{X}}_{t}=\left[{\hat{x}}_{t},z_{t},\psi_{t}\right]$ in a 2D plane, where ${\hat{x}}_{t}$ and $z_{t}$ denote the end-effector position and $\psi_{t}$ denotes the end-effector orientation. The elbow joint is capable of deflection angles $\theta_{\min}\leq \theta \leq \theta_{\max}$ , where $\theta_{\min}$ and $\theta_{\max}$ are the (mechanical) limits. Figure 3 illustrates when ψ is positive or negative.

Generally, there are three possible robot configurations: downward bending, upward bending, and straight configuration. The distinction between the different cases is relevant as the equations representing the geometric constraints are slightly different for each case. $\psi_{t}$ describes the end-effector orientation of the projected target pose ${\hat{X}}_{t}=\left[{\hat{x}}_{t},z_{t},\psi_{t}\right]$ and must be compared with $\psi_{S}$ , which is computed using Equation 6.

$$\psi_{S}=\text{atan}2\left({\hat{x}}_{t},z_{t}\right).$$ (6)

In the straight configuration, the distal part $l_{d}$ , the bending part $l_{b}$ , and the end-effector $l_{e}$ are aligned as displayed by robot 3 in Figure 3. It should be noted that in this case, the bending part is straight and not bent, and $\theta =\psi_{S}=\psi_{t}$ . The case distinction is carried out according to Equation 7.

$$\psi_{t}\left\{\begin{array}{cc} <\psi_{S},\; & \text{downward bending}\\ =\psi_{S},\; & \text{straight configuration }\\ >\psi_{S},\; & \text{upward bending} \end{array}\right..$$ (7)

### 2.3 Set of equations

For both downward and upward bending, the set of equations representing the geometric constraints is provided in Equations 8–11.

$$r^{2}=a^{2}+b^{2},$$ (8)

$$tan\left(\theta \right)=\frac{b}{a},$$ (9)

$$r^{2}=c^{2}+d^{2},$$ (10)

$$tan\left(\psi \right)=\frac{c}{d}.\;$$ (11)

In Sections 2.4, 2.5, the placeholders a, b, c, and d are substituted by the arc parameters $j=\left[{\hat{x}}_{0},z_{0},\theta ,r\right]$ . ${\hat{x}}_{0}$ and $z_{0}$ denote the center coordinates of the projected arc in the global x–z-plane, θ describes the deflection angle of the elbow joint, and r is the radius of the arc. After substitution of the placeholders, the set of equations will only depend on known hardware design parameters (i.e., $l_{b}$ and $l_{e}$ ) and the arc parameters j . This allows for the numerical solution of the arc parameters j , which in turn can be used to compute the generalized coordinates **q**.

### 2.4 Downward bending

**FIGURE 4:**
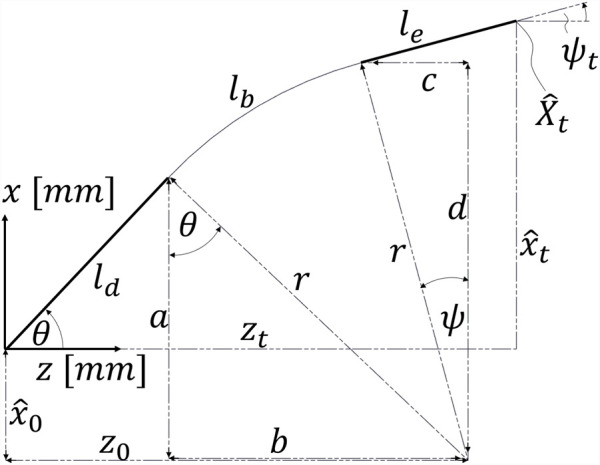
Visualizing the arc parameters $j=\left[{\hat{x}}_{0},z_{0},\theta ,r\right]$ for downward bending; i.e., $\psi_{t}<\psi_{S}$ .

For $\psi_{t}<\psi_{S}$ , the robot performs downward bending. Notably, in downward bending, $\psi_{t}$ may be either positive $\left(0{}^{\circ}<\psi_{t}<\psi_{S}\right)$ or negative $\left(\psi_{t}<0{}^{\circ}\right)$ . In Figure 4, the arc parameters $j=\left[{\hat{x}}_{0},z_{0},\theta ,r\right]$ are shown for downward bending.

Equations 8–11 can be trivially derived from the Pythagorean and angular relationships, as displayed in Figure 4. For downward bending, the placeholders a, b, c, and d, defined in the set of equations representing the geometric constraints (Equations 8–11), must be substituted as follows:

$$a=-{\hat{x}}_{0}+sin\left(\theta \right)\cdot l_{d},$$ (12)

$$b=z_{0}-cos\left(\theta \right)\cdot l_{d},$$ (13)

$$c=z_{0}-z_{t}+cos\left(\psi_{t}\right)\cdot l_{e},$$ (14)

$$d=-{\hat{x}}_{0}+{\hat{x}}_{t}-sin\left(\psi_{t}\right)\cdot l_{e}.$$ (15)

In the substituted set of equations, only the arc parameters $j=\left[{\hat{x}}_{0},z_{0},\theta ,r\right]$ are unknown.

### 2.5 Upward bending

**FIGURE 5:**
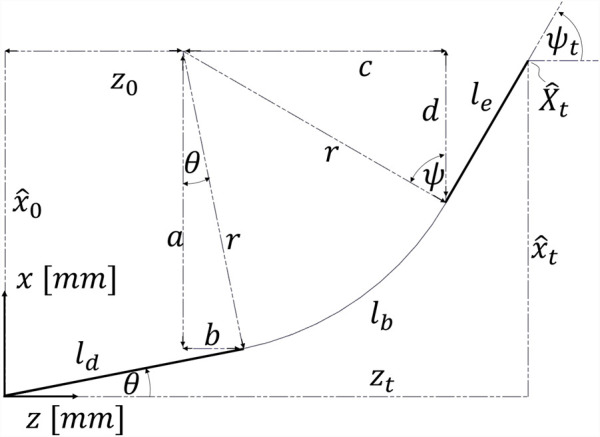
Visualizing the arc parameters $j=\left[{\hat{x}}_{0},z_{0},\theta ,r\right]$ for upward bending; i.e., $\psi_{t}>\psi_{S}$ .

It is evident that the same straightforward geometric relationships described in the set of equations also apply to upward bending. For $\psi_{t}>\psi_{S}$ , the robot performs upward bending as shown in Figure 5. For upward bending, the placeholder variables defined in the set of equations representing the geometric constraints (Equations 8–11) must be substituted as follows:

$$a={\hat{x}}_{0}-sin\left(\theta \right)\cdot l_{d},$$ (16)

$$b=cos\left(\theta \right)\cdot l_{d}-z_{0},$$ (17)

$$c=z_{t}-cos\left(\psi_{t}\right)\cdot l_{e}-z_{0},$$ (18)

$$d={\hat{x}}_{0}-{\hat{x}}_{t}+sin\left(\psi_{t}\right)\cdot l_{e}.\;$$ (19)

The steps so far can be summarized as follows:

The given target pose $X_{t}$ in 3D is projected to 2D, i.e., ${\hat{X}}_{t}$ . Then, it needs to be determined in which configuration the robot can reach ${\hat{X}}_{t}$ , i.e., downward or upward bending. Depending on the configuration, the placeholders in the set of equations are substituted accordingly. In the substituted set of equations, only the arc parameters $j=\left[{\hat{x}}_{0},z_{0},\theta ,r\right]$ are unknown.

### 2.6 (Almost) Straight configuration

As $\psi_{t}$ approaches $\psi_{S}$ , the bending becomes less pronounced, and the radius r increases significantly, as described by Equation 20.

$$\left(\psi_{t}\to \psi_{S}\right)\Rightarrow \left(r\to \infty \right).$$ (20)

**FIGURE 6:**
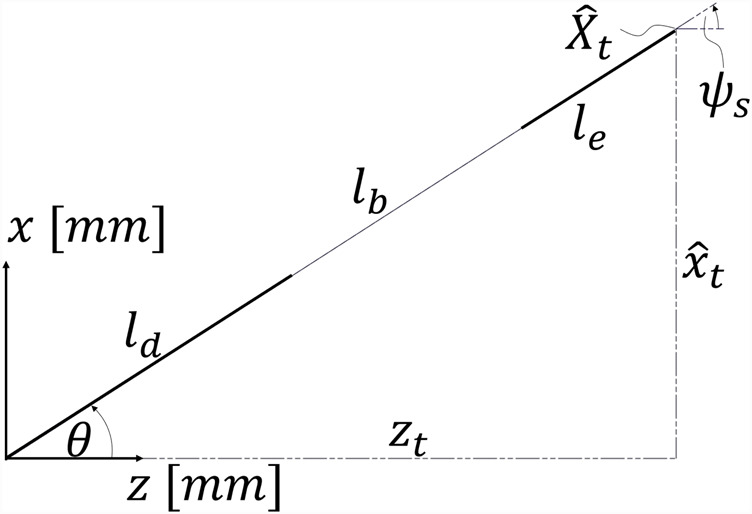
Visualizing the straight configuration for $\theta =\psi_{t}=\psi_{s}$ .

Finding a numerical solution where the variables approach infinity is unfeasible. Thus, to avoid divergence issues, in cases where the robot is straight or almost straight (i.e., $|\psi_{t}-\psi_{S}|<$ 1°), the robot is regarded as being in a straight configuration. For $\psi_{t}=\psi_{S}=\theta$ , the robot performs no bending, as visualized in Figure 6; hence, there is no need to find arc parameters j using Powell’s hybrid method.

Instead, the generalized coordinate $l_{b}$ is computed analytically according to Equation 21.

$$l_{b}=\sqrt{{\left({\hat{x}}_{t}-sin\left(\psi_{t}\right)\cdot \left(l_{e}+l_{d}\right)\right)}^{2}+{\left(z_{t}-cos\left(\psi_{t}\right)\cdot \left(l_{e}+l_{d}\right)\right)}^{2}}.$$ (21)

Thus, in case of a straight configuration, the generalized coordinates are $q=\left[l_{b},\Delta l_{b}=0\;mm,\alpha ,\theta =\psi_{t}\right]$ .

Reverting to a linear equation for the (almost) straight configuration ensures an extremely low runtime while maintaining acceptable accuracy. For the upward and downward bending scenarios, the set of equations is solved using Powell’s hybrid method, as described in the next subsection.

### 2.7 NSGC using Powell’s hybrid method

Powell’s hybrid method (Powell, 1970), also known as Powell’s dog-leg method, is an iterative optimization algorithm used to solve systems of nonlinear equations. It is designed to find the roots of systems of N nonlinear functions in N variables. In this article, it is used to numerically solve the set of nonlinear equations defined in Equations 8–11, where the placeholder variables a, b, c, and d are defined in Equations 12–15 for downward bending and in Equations 16–19 for upward bending. The objective function $f_{{\hat{X}}_{t}}$ is stated in Equation 22.

$$f_{{\hat{X}}_{t}}\left(j\right)=f_{{\hat{X}}_{t}}\left({\hat{x}}_{0},z_{0},\theta ,r\right)=0,$$ (22)

where $f_{{\hat{X}}_{t}}$ represents the substituted set of equations and ${\hat{x}}_{0},z_{0},\theta ,r$ represent the unknown arc parameters j . Now, Powell’s hybrid method is briefly outlined and applied to the equations representing the geometric constraints.

Initialization:

- Set the initial guess $j_{0}=\left[{\left({\hat{x}}_{0}\right)}_{0},{\left(z_{0}\right)}_{0},{\left(\theta \right)}_{0},{\left(r\right)}_{0}\right]$ .
- Define the trust region radius Δ .
- Initialize the scaling matrices $D_{1}$ and $D_{x}$ if necessary, where $D_{1}$ scales the equations and $D_{x}$ scales the variables (Chen and Stadtherr, 1981).

Iteration:

1) Evaluate the function $f_{{\hat{X}}_{t}}\left(j_{k}\right)$ and the Jacobian matrix $J\left(j_{k}\right)$ or its approximation B (Chen and Stadtherr, 1981).
2) Calculate the Gauss–Newton step using the Jacobian as defined in Equation 23

$$p_{N}=-J{\left(j_{k}\right)}^{-1}f_{{\hat{X}}_{t}}\left(j_{k}\right),$$ (23)

or its approximation as defined in Equation 24

$$p_{N}=-B^{-1}f_{{\hat{X}}_{t}}\left(j_{k}\right).$$ (24)

If B is used, it is often an approximation of the Jacobian, such as from a quasi-Newton method (Chen and Stadtherr, 1981).

3) Check whether the Gauss–Newton step is within the trust region as defined in Equation 25

$$\left\|p_{N}\right\|\leq \Delta .$$ (25)

Then, set $p=p_{N}$ and proceed to update the solution.

4) Calculate the Cauchy point: If the Gauss–Newton step is outside the trust region $\left(\left\|p_{N}\right\|>\Delta \right)$ , calculate the steepest descent direction (Cauchy point) in scaled coordinates in scaled coordinates as defined in Equation 26:

$$g=-D_{1}B^{T}D_{1}^{-1}f_{{\hat{X}}_{t}}\left(j_{k}\right).$$ (26)

The Cauchy point is then found by minimizing the quadratic function along the steepest descent direction as defined in Equation 27:

$$Q\left(\mu g\right)=\frac{1}{2}{\left\|D_{1}\left(f_{{\hat{X}}_{t}}\left(j_{k}\right)+\mu Bg\right)\right\|}^{2}.$$ (27)

The multiplier μ is chosen to minimize Q(μg) , which is a quadratic function of μ as stated in Equation 28::

$$\mu =-\frac{f_{{\hat{X}}_{t}}{\left(j_{k}\right)}^{T}Bg}{g^{T}B^{T}Bg}.$$ (28)

The Cauchy point is then given by $p_{S}=\mu g$ .

5) Determine the dog-leg step:
  - If $\left\|p_{S}\right\|\leq \Delta$ , set $p=p_{S}$ .
  - If $\left\|p_{S}\right\|>\Delta$ , find the intersection of the dog-leg path with the trust region boundary as defined in Equation 29:

$$p=\alpha p_{N}+\left(1-\alpha \right)p_{S},$$ (29)

where α is found such that ‖p‖=Δ .

6) Update the solution: $j_{k+1}=j_{k}+p$.
7) Check for convergence: Evaluate the function at the new point and check whether the tolerance threshold or the maximum number of steps criterion is met. If either condition is satisfied, stop the iteration. Otherwise, repeat the process.

This method ensures global convergence by combining the Gauss–Newton direction with the steepest descent direction within a trust region, making it robust for the set of equations representing the geometric constraints.

### 2.8 Initial guesses

To ensure that NSGC finds a solution, it is necessary to provide multiple initial guesses $x_{0}$ for the algorithm to iterate through. In practice, it is expedient to establish different initial guesses for slight bending (e.g., $|\psi_{t}-\psi_{S}|\leq$ 15°) and strong bending (e.g., $|\psi_{t}-\psi_{S}|>$ 15°). This approach allows the NSGC algorithm to avoid iterating through all the initial guesses, bypassing those that are potentially ill-suited for the current bending. For very slight bending (e.g., $|\psi_{t}-\psi_{S}|<$ 1°), the robot is considered to be in a straight configuration, as detailed in Section 2.6.

### 2.9 Projection with elbow joint constraints

If the projection of the target pose is always performed positively, such that $\text{proj}\left(X_{t}\right)={\hat{X}}_{t}=\left[{\hat{x}}_{t},z_{t},\psi_{t}\right]$ with ${\hat{x}}_{t}=\sqrt{x_{t}^{2}+y_{t}^{2}}$ according to Equation 4, certain poses become unreachable due to mechanical joint limits. This issue is illustrated in Figure 2 for robot 2; if the projection $\hat{X}t$ were carried out positively (i.e., with a rotation of $\alpha_{2}=25{}^{\circ}$ ) and assuming constant curvature of the bending section, the elbow joint would need to bend downward beyond its lower bound $\theta <\theta_{\min}$ , thus violating the joint constraint. Consequently, the projection must instead be performed negatively with ${\hat{x}}_{t}=-\sqrt{x_{t}^{2}+y_{t}^{2}}$ and $\alpha_{2}=-155{}^{\circ}$ .

As mentioned in Section 2.1, it is not known *a priori* whether a given target pose $X_{t}$ should be projected positively or negatively. The limit of the feasible end-effector orientation ${\bar{\psi }}_{t}$ depends on both the target position $\left({\hat{x}}_{t},z_{t}\right)$ and the elbow joint constraint. For the case $\theta_{\min}=0{}^{\circ}$ , the boundary of the reachable orientation is defined using the following equations (Equations 30–32):

$$r={\hat{x}}_{0},$$ (30)

$$r^{2}={\left(r-{\hat{x}}_{t}+sin\left(\bar{\psi }\right)\cdot l_{w}\right)}^{2}+{\left(z_{t}-l_{d}-cos\left(\bar{\psi }\right)\cdot l_{w}\right)}^{2},$$ (31)

$$\begin{array}{l} \begin{array}{cc} \bar{\psi }=atan2 & \left(\left(z_{t}-l_{d}-cos\left(\bar{\psi }\right)\cdot l_{w}\right),\right.\\ & \left.\left(r-{\hat{x}}_{t}+sin\left(\bar{\psi }\right)\cdot l_{w}\right)\right) \end{array}. \end{array}$$ (32)

**FIGURE 7:**
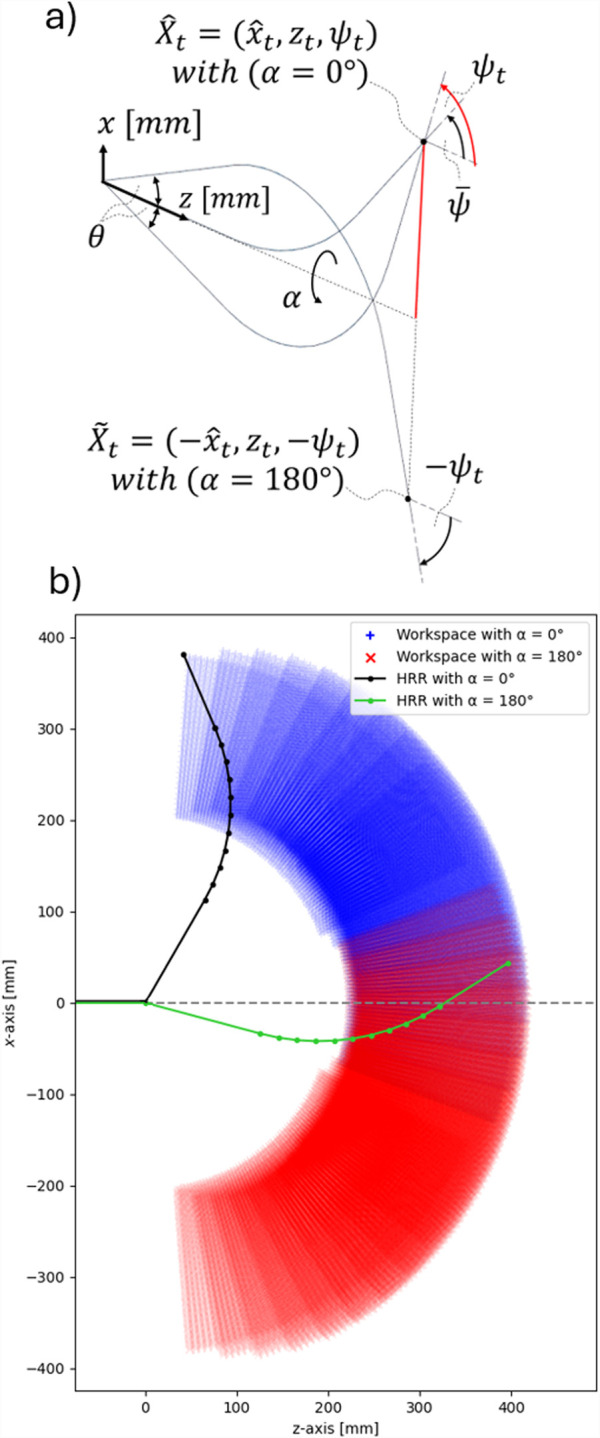
**(a)** Utilizing α to reach target poses with $\psi_{t}>\bar{\psi }$ . **(b)** Workspace enlargement by rotating the robot about its main axis (e.g., α= 180°).

Here, the variables ${\hat{x}}_{0}$ , r , and $\bar{\psi }$ are unknowns. After solving this system, $\bar{\psi }$ is compared to the target end-effector orientation $\psi_{t}$ to determine whether a positive or negative projection is appropriate. If $\psi_{t}\leq \bar{\psi }$ , a positive projection is feasible; otherwise, the projection must be carried out negatively. In the latter case, the robot must rotate about its main axis and approach the target from the $-\hat{x}$ direction, thereby staying within the joint constraint $\theta \geq \theta_{\min}=0{}^{\circ}$ and achieving ${\hat{X}}_{t}=\left[{\hat{x}}_{t},z_{t},\psi_{t}\right]$ with $\psi_{t}>\bar{\psi }$ , as illustrated in Figure 7a).

This rotation about the main axis significantly enlarges the workspace, as shown in Figure 7b. When a target pose is only reachable via this axial rotation, the projected pose must be transformed as ${\hat{X}}_{t}=\left[{\hat{x}}_{t},z_{t},\psi_{t}\right]\to {\tilde{X}}_{t}=\left[-{\hat{x}}_{t},z_{t},-\psi_{t}\right]$ . This corresponds to the rotated configuration. In that case, α [Equation 5] has to be increased by 180°. The pose ${\tilde{X}}_{t}$ is then used as the target for inverse kinematics and solved using Powell’s hybrid method.

### 2.10 False positives and convergence conditions

**FIGURE 8:**
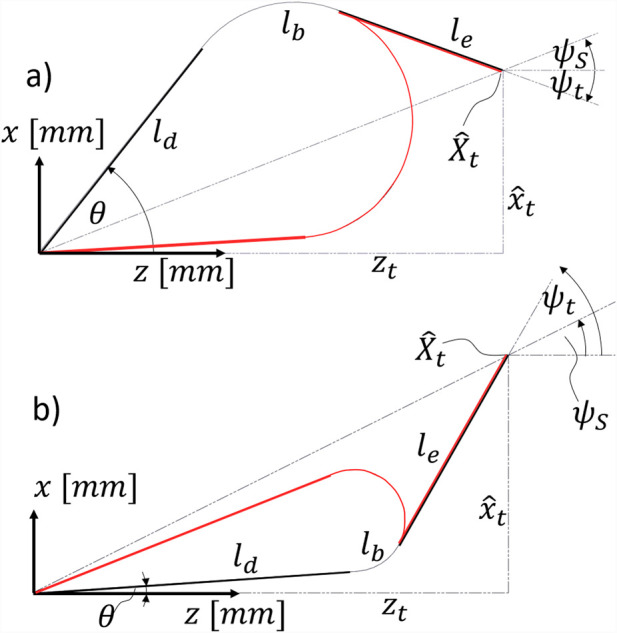
Black robots are correct, and the red robots are false positives. **(a)** Downward bending: instead of having $\theta >\psi_{S}$ and then a downward bending, this NSGC solution has $\theta \;<\;\psi_{S}$ and then an upward bending. **(b)** Upward bending: the numerical solution provides a downward bending robot such that the bending part $l_{b}$ and end-effector $l_{e}$ are tangents. However, this solution does not represent the physical nature of the robot and needs to be filtered out.

NSGC can provide false positives that are mathematically correct but do not represent the physical robot. The predominant cases of false positives are shown in Figure 8.

It must be ensured that the algorithm does not accept a false positive as a numerical solution. Thus, for each solution is checked against the following three convergence conditions:

- 0° $\overset{\leq }{!}\theta \overset{\leq }{!}$ 60°.
- If $\psi_{t}<\psi_{S}$ (i.e., downward bending), then $\theta \overset{>}{!}\psi_{S}$ (Figure 8a).
- If $\psi_{t}>\psi_{S}$ (i.e., upward bending), then $sin\left(\theta \right)\cdot l_{d}\overset{<}{!}{\hat{x}}_{T}-sin\left(\psi \right)\cdot l_{e}$ (Figure 8b).

It should be noted that for upward bending, condition III is stronger than the statement “if $\psi_{t}>\psi_{S}$ , then $\theta \overset{!}{<}\psi_{S}$ .” As shown in Figure 8b, there exists a case where $\theta <\psi_{S}$ , but the solution is false. Such a strong formulation is not possible for downward bending, i.e., condition II.

### 2.11 Getting from numerical solution to generalized coordinates

w is a design parameter of the robot that describes the distance between the backbone and the imaginary centerline. This distance is identical to the distance between the connectors and the imaginary centerline. The radius r denotes the radius of the imaginary centerline, as shown in Figure 1.

Using the numerically computed deflection angle θ and radius r , the length of the bending part (i.e., $l_{b}$ ) and the difference between $l_{b}$ and the length of the backbone (i.e., $\Delta l_{b}$ ) are computed according to Equations 33, 34 for downward bending.

$$l_{b}=\left(r-w\right)\cdot \left(|\psi |+\theta \right),$$ (33)

$$\Delta l_{b}=\left(r+w\right)\cdot \left(|\psi |+\theta \right)-l_{b}.\;$$ (34)

$l_{b}$ and $\Delta l_{b}$ for upward bending are computed according to Equations 35, 36.

$$l_{b}=\left(r+w\right)\cdot \left(\psi -\theta \right),$$ (35)

$$\Delta l_{b}=\left(r-w\right)\cdot \left(\psi -\theta \right)-l_{b}.\;$$ (36)

To derive the resulting end-effector pose based on the NSGC solution for the generalized joint coordinates $q=\left[l_{b},\Delta l_{b},\alpha ,\theta \right]$ , q has to be plugged into the forward kinematic model defined by Equation 2. The resulting end-effector pose can then be compared to the target pose, with the target pose $X_{t}$ .

### 2.12 Overview of NSGC

In summary, the final NSGC algorithm is expressed in Algorithm 2. Here, $X_{t}$ denotes the original target pose in 3D, ${\hat{X}}_{t}$ denotes the 2D projection, and ${\tilde{X}}_{t}$ denotes a rotation thereof.

Algorithm 2 NSGC.

1: **Receive** $X_{t}\leftarrow \left[x_{t},y_{t},z_{t},\psi_{t}\right]$ ⊳ target pose in 3D space
2: **Project** it into 2D space ${\hat{X}}_{t}\leftarrow \left[{\hat{x}}_{t},z_{t},\psi_{t}\right]$ [Equation 4]
3: **Compute** α [Equation 5]
4: **Determine** robot configuration [Equations 6, 7]
5: **for** i=1→n **do**
6: $j_{0}^{i}\leftarrow \left[{\left({\hat{x}}_{0}\right)}_{0},{\left(z_{0}\right)}_{0},{\left(\theta \right)}_{0},{\left(r\right)}_{0}\right]$ ⊳ initial guesses
7: **end for**
8: **if** robot configuration = downward bending, **then**
9:  **Define** a set of equations $f_{{\hat{X}}_{t}}$ [Equations 8–11] with [Equations 12–15]
10:  **while** convergence conditions are not met **do**
11: $j\leftarrow \text{PowellSolve}\left(f_{{\hat{X}}_{t}},j_{0}^{i}\right)$ [Equation 22]
12: $j_{0}^{i}\leftarrow j_{0}^{i+1}$

13:  **end while**
14:  **Compute** $l_{b}$ and $\Delta l_{b}$ [Equations 33, 34].
15: **else if** robot configuration = upward bending, **then**
16:  **Define** a set of equations $f_{{\hat{X}}_{t}}$ [Equations 8–11] with [Equations 16–19]
17:  **while** Convergence conditions are not met **do**
18: $j\leftarrow \text{PowellSolve}\left(f_{{\hat{X}}_{t}},j_{0}^{i}\right)$ [Equation 22]
19: $j_{0}^{i}\leftarrow j_{0}^{i+1}$

20:   **if** no convergence, **then**
21:    **while** Convergence conditions are not met **do**
22: ${\tilde{X}}_{t}\leftarrow \left[-{\hat{x}}_{t},z_{t},-\psi_{t}\right]$ ⊳ rotate by α= 180°
23: $j\leftarrow \text{PowellSolve}\left(f_{{\tilde{X}}_{t}},j_{0}^{i}\right)$ [Equation 22]
24: $j_{0}^{i}\leftarrow j_{0}^{i+1}$

25:    **end while**
26:    **Update** α←α+π

27:   **end if**
28:  **end while**
29:  **Compute** $l_{b}$ and $\Delta l_{b}$ [Equations 35, 36]
30: **else**                  ⊳ Straight configuration
31:  **Compute** $l_{b}$ [Equation 21]
32: $\Delta l_{b}\leftarrow 0$

33: **end if**
34: **Send** $q=\left[l_{b},\Delta l_{b},\alpha ,\theta \right]$ to motors

**FIGURE 9:**
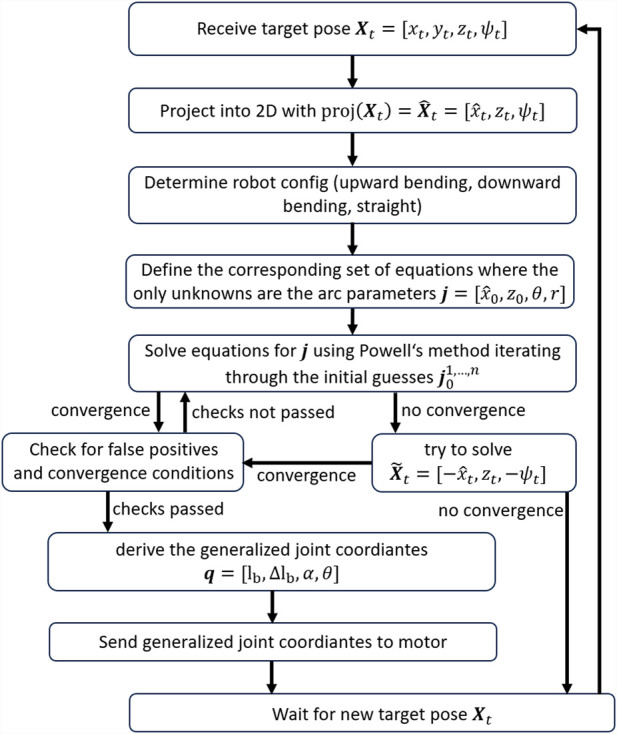
NSGC flowchart.

The flowchart in Figure 9 visualizes the individual steps of the NSGC algorithm.

## 3 In silico validation

The proposed NSGC algorithm was implemented and compared with four state-of-the-art IKMs following a helical trajectory: two basic Jacobian-based models with k-factor values of 0.4 and 0.5, one Levenberg–Marquardt (LM) implementation with adaptive damping, and one quadratic programming approach. The LM technique is also known as damped least squares in optimization. A k-factor of 0.5 was the highest value that still allowed all target poses to be achieved. The comparison included the accuracy, median, mean, range, IQR, and standard deviation of the convergence times for 200 target poses $X_{t}=\left[x_{t},y_{t},z_{t},\psi_{t}\right]$ along the helix.

### 3.1 Convergence time and behavior

**FIGURE 10:**
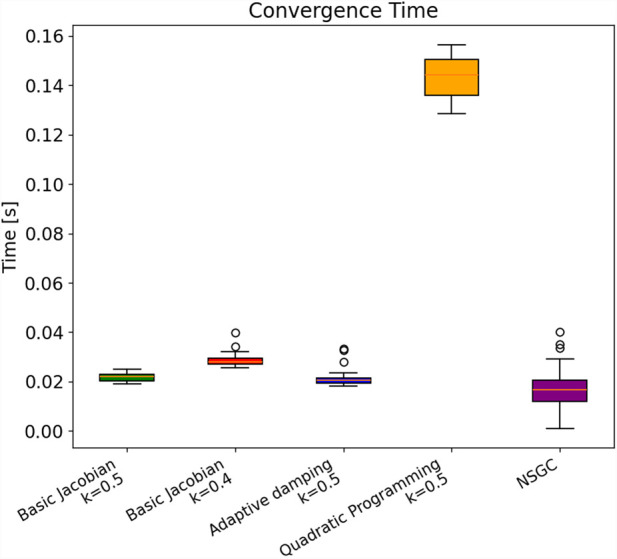
Average time to solve 1,000 randomly generated 3D poses.

The convergence times for all five IKMs are visualized in Figure 10 and summarized in Table 1. The mean convergence times of basic Jacobians and the adaptive damping LM IKMs are very similar to each other (in the range of 21 ms29 ms ), which is significantly faster than 100 ms needed for real-time applications. NSGC achieves a mean convergence time of 16 ms , which is approximately 24% faster than the fastest competing IKM (i.e., the adaptive damping IKM). As shown in Figure 10, the quadratic programming approach converges with a mean time of 143 ms , which is notably slower than that of the four other models. Therefore, the quadratic programming IKM is not usable for real-time applications.

**TABLE 1:** Overview of simulation results.

Metric	Basic Jacobian k = 0.5	Basic Jacobian k = 0.4	Adaptive damping k = 0.5	Jacobian with QP	NSGC
Successfully reached poses [%]	100	100	100	100	100
Median time [ms]	22	28	21	144	17
Mean time [ms]	22	29	21	143	16
Range [ms]	6	14	15	28	39
IQR [ms]	3	2	2	15	9
Standard deviation [ms]	1.55	2.39	2.93	8.01	7.94

It has to be noted that the spread of convergence time of NSGC (39 ms) is notably higher than that of all other IKMs, ranging from 6 ms−28 ms . This large spread of 39 ms can be explained by the necessary iteration through many initial guesses in case the previous initial guess did not lead to a convergence. However, even the longest measured convergence time was only ≈40 ms . In contrast, in some cases, the very first initial guess led to convergence, which resulted in an extremely fast convergence time of less than 5 ms , outperforming all other models. IQR stands for interquartile range and measures statistical dispersion, specifically representing the spread of the middle 50% of a dataset.

**FIGURE 11:**
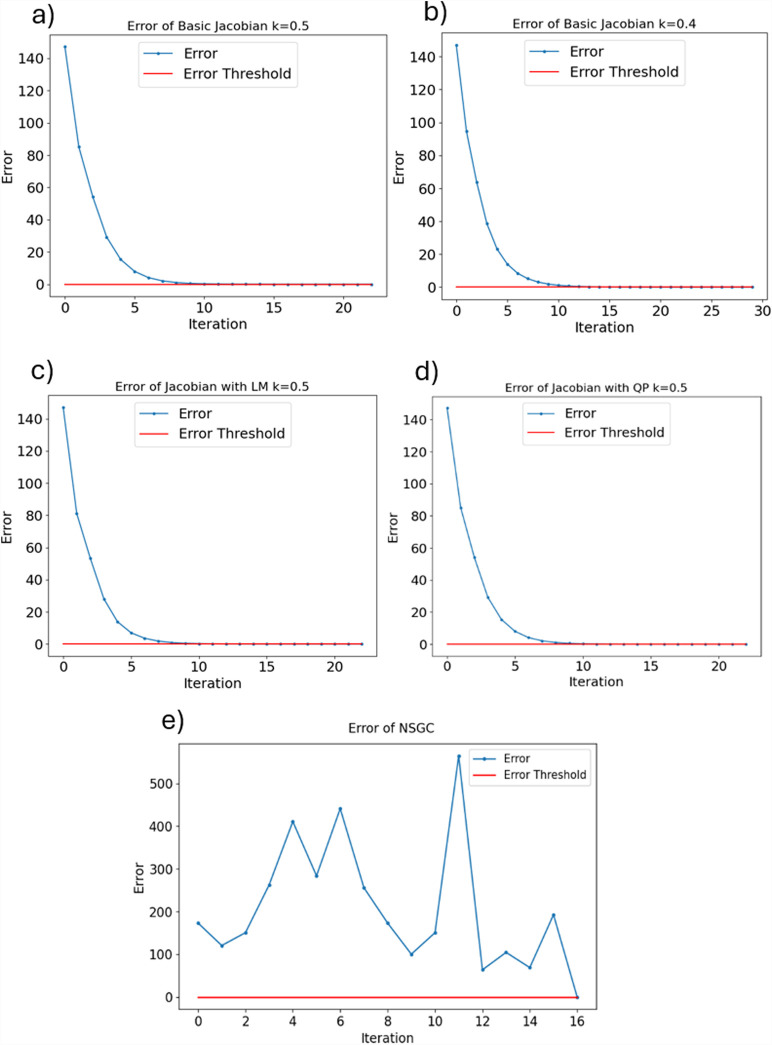
Comparison of convergence behavior of NSGC vs. Jacobian-based IKMs with an error threshold of 1e-5. **(a)** the basic jacobian IKM with k=0.5 converges after 22 steps. **(b)** the basic jacobian IKM with k=0.4 converges after 29 steps. **(c)** the LM IKM converges after 22 steps. **(d)** the QP IKM converges after 22 steps, but each step takes significantly longer than using the basic Jacobian-based IKM. **(d)** NSGC needs 16 steps.

The convergence behavior of NSGC is displayed in Figure 11. As observed, NSGC iterates through different initial guesses until it finds the correct solution for the set of equations defined in Section 2.3. It is evident that NSGC’s convergence behavior is fundamentally different from that of all other IKM approaches (Figure 11) as there is no convergence in the sense that the error progressively approaches 0. Rather, the convergence of an iteration is completely independent of the previous iteration’s convergence, as it depends solely on the initial guess. Note that the convergence time is not only influenced by the number of iterations until convergence but also by the time needed per iteration. Generally, NSGC and the quadratic programming approach need more time per iteration.

### 3.2 Memory footprint and computational complexity

The NSGC algorithm, implemented in Python, exhibits a moderate memory footprint and computational complexity.

#### 3.2.1 Memory footprint

The memory usage of NSGC is primarily driven by the number of initial guesses for solving the nonlinear system, with each being a 4-dimensional vector $\left(z_{0},{\hat{x}}_{0},\theta ,r\right)$ stored in the initial_guesses list. Let n be the number of such guesses. Each guess is a list of four floating-point numbers (8 bytes each), so the memory for the guesses alone is O(n) , which is typically a few hundred bytes to a few kilobytes. Additional memory is consumed by temporary arrays during forward kinematics computation, but due to Python’s garbage collection and the linear chaining of matrices, peak memory usage remains modest—typically only a few megabytes.

#### 3.2.2 Computational complexity

The dominant cost arises from numerically solving a nonlinear system of four equations with four unknowns using Powell’s method. For each initial guess, it performs iterative updates until convergence, which typically takes k iterations per guess, with each requiring evaluation of the function and Jacobian approximation.

Let n be the number of initial guesses and k be the number of iterations per call to Powell’s method.

The overall complexity becomes

$$O\left(n\cdot k\cdot c\right),$$

where c is the cost of evaluating the nonlinear system (consisting of elementary operations). Since most branches terminate early upon finding a valid solution, the average-case complexity is significantly lower than the worst-case complexity.

#### 3.2.3 Practical performance

Empirical results indicate solution times in the range of tens of milliseconds on a modern CPU, depending on the initial guess set and configuration. Memory remains bounded and scales linearly with the resolution and number of guesses, making the algorithm suitable for embedded applications with moderate resources.

### 3.3 Robustness to noise, initial guess sensitivity, and singularities

The NSGC algorithm demonstrates high robustness in practical inverse kinematic scenarios but retains certain sensitivities that merit discussion.

#### 3.3.1 Robustness to noise

When using NSGC, the user has free choice of the position $\left(x_{t},y_{t},z_{t}\right)$ defined in the global coordinate system (CS) and free choice of the orientation $\left(\psi_{t}\right)$ defined as the rotation about the $y_{\varepsilon }$ -axis of local end-effector CS. Noise in these four target dimensions is inconsequential, as the noisy target pose $X_{t,noise}=\left[x_{t}+\eta_{1},y_{t}+\eta_{2},z_{t}+\eta_{3},\psi_{t}+\eta_{4}\right]$ will be solved by NSGC, with η representing random noise. The remaining two orientations about the local $x_{\varepsilon }$ - and $z_{\varepsilon }$ -axis of the end-effector are always set to 0, indicating that noise in these target dimensions will also not influence the convergence behavior.

#### 3.3.2 Sensitivity to initial guess

The choice of initial guesses significantly impacts both convergence success and computational efficiency. While NSGC employs a coarse angular sweep strategy to sample possible solution regions, poorly chosen or sparse guess sets can lead to missed solutions or divergence. Empirical evidence suggests that a resolution of approximately 10° for the initial guess of ${\left(\theta \right)}_{0}$ and approximately 30 mm for the initial guess of ${\left({\hat{x}}_{0}\right)}_{0},{\left(z_{0}\right)}_{0}$ and ${\left(r_{0}\right)}_{0}$ provides a good trade-off between coverage and runtime. Due to the forgiving nature of Powell’s method, it often converges even with suboptimal initial guesses; e.g., $|{\left({\hat{x}}_{0}\right)}_{0}-{\hat{x}}_{0}|>50\;mm$ .

#### 3.3.3 Singularities and degenerate configurations

The algorithm can struggle near geometric singularities, such as when the NiTi segment length $l_{b}$ approaches 0. The most effective mitigation strategy in this case is to either reduce the length of the distal part $l_{d}$ and end-effector $l_{e}$ or to relocate the robot’s point of deployment further away from the workspace so that the bending section is long enough to provide the needed maneuverability. The most common singularity occurs when a straight configuration is approached (typically $|\psi_{t}-\psi_{S}|<$ 1°). This scenario and the appropriate mitigation strategy are described in Section 2.6.

Overall, good initial guesses, checking for false positives, and establishing a straight-line case for divergent bending radii ensure NSGC’s performance, stability, and accuracy considerably.

### 3.4 Simulated case study

As CRs/HRRs are used in endoscopy (Sun and Chen, 2024) and biopsy procedures (Gao et al., 2020), this study uses endoscopic examination of the gallbladder as a case study. During a biopsy, the physician traverses the surface of the organ, and once a suitable location is identified, the biopsy needle is inserted into the tissue.

According to Can et al. (2012), the average gallbladder size is h×w×l=50 mm×50 mm×30 mm . Hence, in this study, a cuboid was used to represent the size of the gallbladder. For this simulated case study, NSGC was used to solve the trajectory of the end-effector traversing the cuboid’s surface. The full simulation video is available in the Supplementary Material.

The following section describes the robotic prototype used for the experimental validation.

## 4 HyNiTi prototype

The robotic prototype is referred to as a hyper-redundant, NiTi-based robot (abbreviated as HyNiTi). The robot’s dexterity stems from its ability to adjust the length and incorporate an elbow joint, enabling multiple orientations at the same position (Fritsch et al., 2024).

**FIGURE 12:**
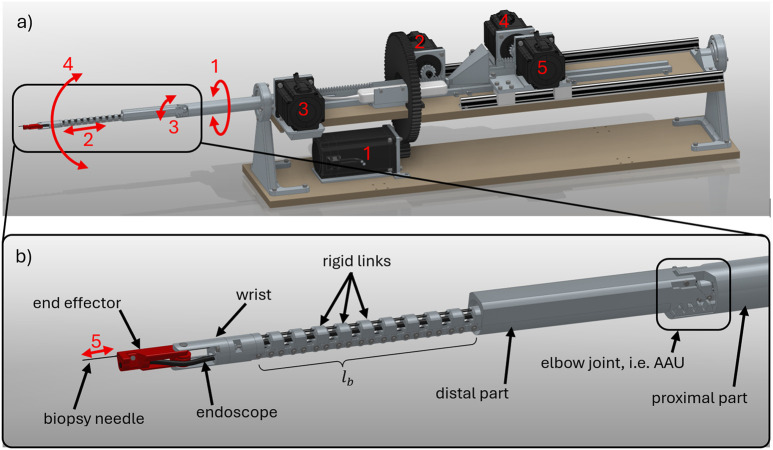
CAD model of HyNiTi: **(a)** complete robot including motors and **(b)** details of HRR; scale: $l_{b}=200\;mm$ in this figure.

The HyNiTi features four motors (motors 1–4 in Figure 12) for robot actuation, resulting in four DoFs of the robot. Additionally, there is one motor (motor 5 in Figure 12) for instrument actuation (i.e., advancing and retracting the biopsy needle). The entire robot is rotated about its main axis by approximately α by motor 1. Motor 2 changes the length of the bending part (i.e., $l_{b}$ ), while motor 3 actuates the elbow joint, causing a deflection in the HRR away from the proximal part’s main axis. Motor 4 changes the length of the backbone (i.e., $\Delta l_{b}$ ), causing the robot to bend. For $\Delta l_{b}<0$ , the robot bends upward, and for $\Delta l_{b}>0$ , the robot bends downward. A clear overview of the relationship between the motor and the DoF is provided in Table 2. Notably, NSGC solves $\alpha ,\theta ,l_{b}$ and $\Delta l_{b}$ . The needle actuation is controlled by the user directly.

**TABLE 2:** Overview of motors and DoF.

Motor	1	2	3	4	5
DoF	α	$l_{b}$	θ	$\Delta l_{b}$	Needle

**FIGURE 13:**
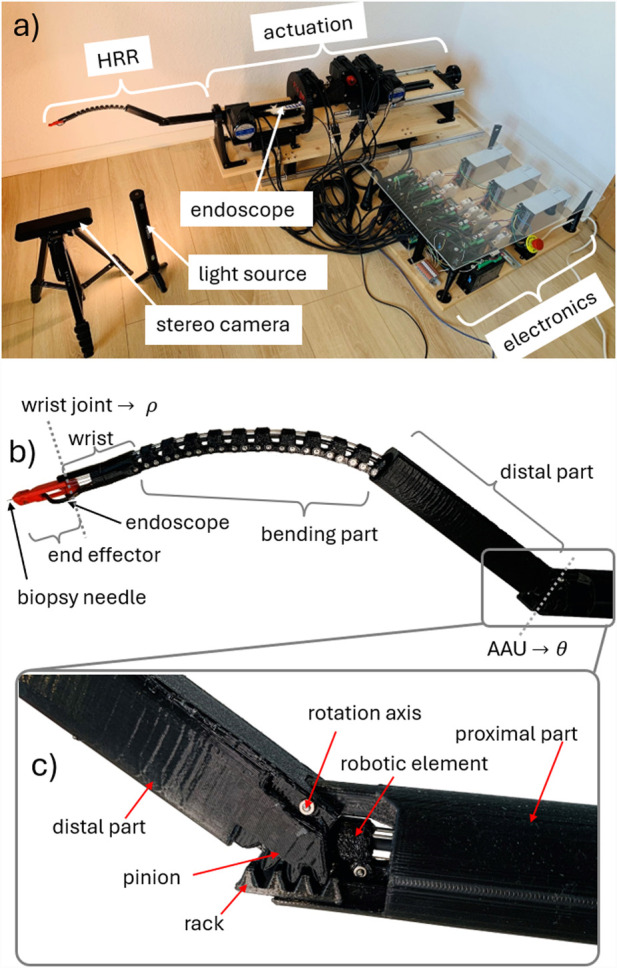
Robotic prototype: **(a)** test setup, **(b)** HRR details, and **(c)** elbow joint for scale: distal part of this prototype is $l_{d}=130\;mm$ long.

The endoscope (K-FLEX-XC1, Karl Storz SE & Co. KG, Tuttlingen, Germany) is routed through the HRR to its end-effector. The endoscope is not directly actuated but complies with the shape of the HRR. The endoscope features a 3 mm outer diameter, a light source, a camera, and a working channel. The entire robot, including the actuation and electronic components, is shown in Figure 13a, whereas Figures 13b, c show the HRR and the elbow joint in more detail.

### 4.1 HRR actuation

HyNiTi is an HRR that relies on backbone actuation. The backbone is made of highly elastic metal, such as superelastic NiTi or spring steel. Unlike tendons, the backbone is resistant to stretching and backlash, resulting in more stable and predictable robotic behavior.

Another advantage of this design is the reduced number of channels within the robotic elements. Tendon-driven systems typically require separate channels for each bending direction (e.g., one tendon for upward bending and another for downward bending). In contrast, the pull–push backbone actuation requires only one channel to facilitate movement in two opposing directions, enabling further miniaturization of the robot.

### 4.2 Elbow joint design

The prototype utilizes an elbow joint that is based on a rack–and-pinion mechanism (Fritsch et al., 2023). The rack is pushed or pulled by a motor (in this case, by motor 3), causing the pinion to rotate about the axis of rotation. The pinion is structurally integrated into the distal part. To mitigate the risk of tilting or jamming of the distal part, the rack and pinion mechanism is designed with redundancy, incorporating a two-sided configuration.

The elbow joint’s deflection angles are in the range of 0° $=\theta_{\min}\leq \theta \leq \theta_{\max}=$ 60°, where $\theta_{\min}$ and $\theta_{\max}$ are the mechanical limits.

## 5 Experimental validation

The NSGC algorithm was tested using the HyNiTi robot. The complete test setup is shown in Figure 13a. A stereo camera (ZED 2i, Stereolabs, San Francisco, United States) was used for the optical measurement of the achieved poses. The depth information enables the localization of points in 3D so that the x, y, and z coordinates can be derived with respect to the camera. The camera is extrinsically calibrated to the global coordinate system, which is located at the robot’s base. Each of the measured points is transformed into the global coordinate system.

In this setup, each stepper motor features an optical incremental encoder, and the drivers handle the PID control internally; thus, there is low-level closed-loop control. The joint position commands are generated using NSGC and then fed to the stepper motor drivers using a microcontroller.

### 5.1 Individual target poses

**FIGURE 14:**
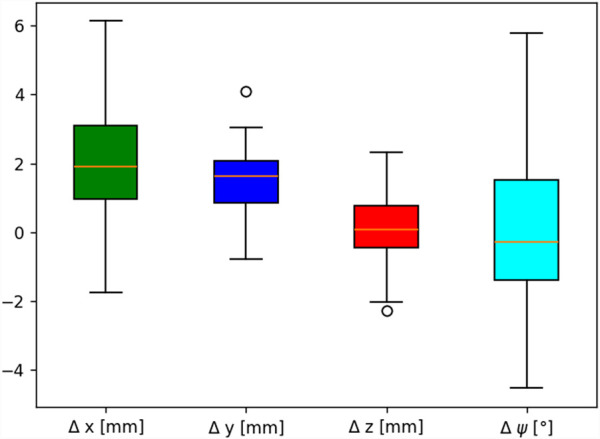
Deviation between the measured pose and target pose for 100 individual target poses in 3D.

A total of 100 target poses $X_{t}$ were solved using NSGC, the generalized coordinates $q=\left[l_{b},\Delta l_{b},\alpha ,\theta \right]$ were calculated and sent to the motors, and the achieved pose was measured and compared to the target pose. A picture including the depth information of each pose was captured using the stereo camera and then analyzed. The analysis was based on two points: the proximal end of the wrist and the distal end of the end-effector. From the coordinates of both points, the achieved orientation about the local $y_{\varepsilon }$ -axis was derived. The differences between the target and measured poses showed good agreement, as shown in Figure 14 and Table 3. RMSE stands for root mean square error.

**TABLE 3:** Statistical error for individual poses.

Error metrics	x	y	z	ψ
RMSE	3.53 mm	3.03 mm	1.70 mm	2.09°
Mean	2.82 mm	2.63 mm	0.42 mm	−0.20°
Standard deviation	2.13 mm	1.52 mm	1.65 mm	2.09°
95% Confidence interval	( 2.40 mm , 3.25 mm )	( 2.33 mm , 2.93 mm )	( 0.09 mm , 0.74 mm )	(-0.62°,0.24°)

As shown in Figure 14, the range of error was the highest for the end-effector angle $\psi_{t}$ , with Δψ ranging from −4 ° to 6° with an RMSE of 2.09°. The RMSE for all three positional coordinates ranged from 1.7 mm along the global z-axis to 3.53 mm along the global x-axis. The mean positional error ranged from 0.42 mm to 2.82 mm . A leading factor contributing to the pose error is the weight of the robot itself, which induces downward bending. The lack of rigidity can be alleviated to some degree by appropriate robot calibration and by increasing the bending section’s stiffness.

### 5.2 Trajectory tracking

**FIGURE 15:**
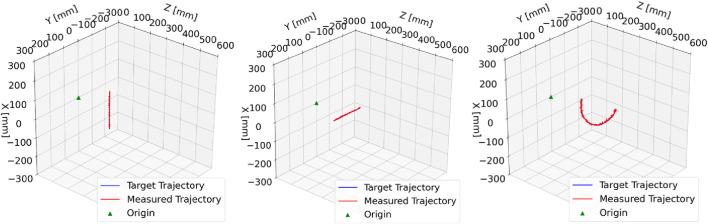
Trajectory tracking: left plot: end-effector tracking along the x-axis from + 100 mm to − 100 mm with 20 s duration; center plot: end-effector tracking along the y-axis from + 100 mm to − 100 mm with 20 s duration; right plot: end-effector tracking along a helix path with 30 s duration.

For the application of CR/HRR technology in the real world, good trajectory tracking performance is crucial. In this experiment, three trajectories were tracked: a linear 200 mm movement in the x-direction, a linear 200 mm movement in the y-direction, and a movement along a 3D helix. The helix was characterized by a radius of 50 mm and a length of 200 mm along the z-axis, as shown in Figure 15. The error metrics are given in Table 4.

The inverse kinematics for each point on the helix was computed using the proposed NSGC algorithm. The orientation at each point was kept constant; in other words, the end-effector reached each pose on the trajectory with the same orientation. In all three cases, the target trajectory was compared against the measured trajectory. During trajectory tracking, an image was captured and analyzed every 0.5 s . The trajectory tracking validation confirms that NSGC can be used for the control of length-extensible CRs/HRRs with an elbow joint since all poses along the path were successfully reached, as shown in Figure 15.

**TABLE 4:** Statistical error for trajectory tracking.

Error metrics	Trajectory along x	Trajectory along y	Helix
RMSE [mm]	4.44	4.47	5.75
Mean [mm]	4.05	4.22	5.31
Standard deviation [mm]	1.80	1.46	2.20
Maximum error [mm]	8.27	7.88	12.05
Minimum error [mm]	0.56	1.19	0.64

The average positional error during trajectory tracking was 4.05 mm along the x-axis and 4.22 mm along the y-axis. These errors are slightly higher than those observed in the measurements of individual points (Section 5.1). This discrepancy is most likely caused by the continuous movement of the robot’s end-effector during trajectory tracking, compared to the stationary end-effector used when measuring individual points. With an average positional error of 5.31 mm , trajectory tracking when following the helical path was considerably less accurate than the linear trajectories along the x- and y-axes. This is most likely due to the added dynamics of the end-effector’s movement along the z-direction. The superposition of movements along all three axes decreases the overall accuracy.

The inaccuracies are due to the mechanical structure of the robot: play in the joints connecting the rigid links in the bending section and a relatively heavy end-effector, which causes low-frequency mechanical vibrations.

### 5.3 Case study: gallbladder biopsy

The HyNiTi prototype, equipped with a biopsy needle and an endoscopic camera (as described in Section 4), was used in this case study to simulate a biopsy on a piece of meat representing the gallbladder. The objective of this case study is to demonstrate NSGC’s effectiveness in real-life applications.

**FIGURE 16:**
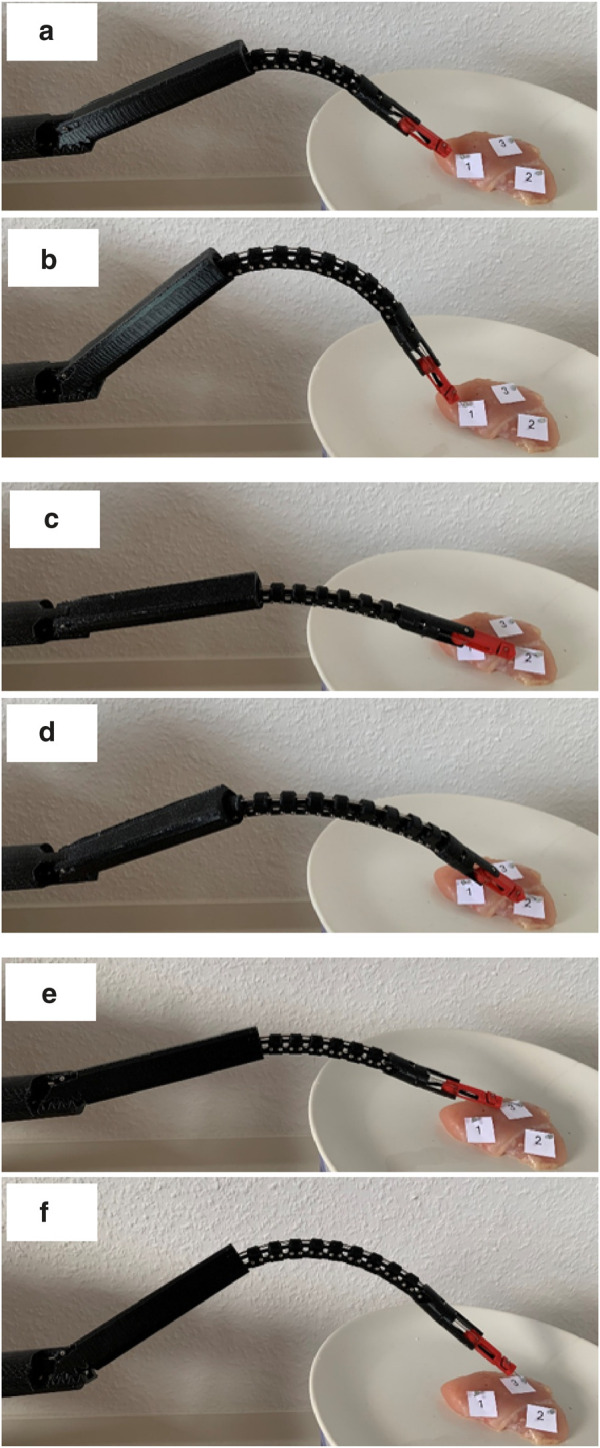
Case study of three target locations on the tissue: **(a)** target position 1 is reached at an angle ψ , which is changed in **(b)**. Target position 2 is located to the right of the robot’s main axis so that the robot rotates in the counterclockwise direction α> 0°. **(c)** and **(d)** show different end-effector orientations ψ at position 2. Target position 3 is located to the left of the robot’s main axis so that the robot rotates in the clockwise direction α< 0°. The target position can also be reached with a variation in ψ , as shown by the difference in orientation between **(e,f)**.

Three distinct target locations were selected on the tissue sample. Position 1 was aligned along the main axis of the proximal region, while positions 2 and 3 were located on the top and bottom surfaces of the tissue, respectively. Reaching these off-axis targets required actuation of the robot’s rotational degree of freedom, denoted as α . As shown in Figure 16, the robot was able to reach all three points on the tissue without any issue.

## 6 Conclusion

In this article, a novel inverse kinematic model termed NSGC was introduced—a numerical solution of a set of equations representing geometric constraints. NSGC is well-suited for length-extensible CRs and HRRs featuring an elbow joint.

A target pose $X_{t}=\left[x_{t},y_{t},z_{t},\psi_{t}\right]$ is given in 3D space, where $x_{t},y_{t}$ , and $z_{t}$ are the global coordinates and $\psi_{t}$ is the end-effector orientation about the local $y_{\varepsilon }$ -axis. $X_{t}$ is projected onto a plane with $\text{proj}\left(X_{t}\right)={\hat{X}}_{t}=\left[{\hat{x}}_{t},z_{t},\psi_{t}\right]$ . The planar arc is described using four equations. The arc parameters $j=\left[{\hat{x}}_{0},z_{0},\theta ,r\right]$ are unknown and solved numerically using Powell’s hybrid method while iterating through appropriate initial guesses. For every potential numerical solution for these arc parameters, it is checked whether convergence conditions I–III are met to eliminate false positives. When all conditions are satisfied, the arc parameters j are used to compute the generalized joint coordinates $q=\left[l_{b},\Delta l_{b},\alpha ,\theta \right]$ , which are then sent to the motors. *In silico* validation shows that NSGC solves the given target poses in 16 ms on average and has a median convergence time of 17 ms . Hence, NSGC can be used for real-time applications. NSGC is 24% faster than the next fastest IKM (Jacobian-based with adaptive damping). Therefore, NSGC could be used in scenarios where minimizing latency between the user input (defining the target pose) and the robot’s movement is critical.

The HRR prototype relied on backbone actuation, which proved to work reliably and without any backlash or elongation compared to tendon actuation. The elbow joint allowed for precise and repeatable deflection of the HRR away from the proximal part’s main axis. Changing the orientation at the same position was enabled by the combination of the elbow joint and the length adjustment of the HRR.

The limitation of NSGC is the narrow range of kinematic structures for which it can be used; the discussed CR/HRR structure with length-adjustment capabilities and an elbow joint can only achieve a position in 3D space and with one orientation about the local $y_{\varepsilon }$ -axis. For such robots, the end-effector orientation about the local $x_{\varepsilon }$ - and $z_{\varepsilon }$ -axes are necessarily 0°. This is the reason why NSGC is a good fit for such kinematics. Incorporating additional orientations about the $x_{\varepsilon }$ - and $z_{\varepsilon }$ -axes of the end-effector poses a challenge since in that case, the 2D projection does not work as elegantly as in the addressed scenario.

In the current trajectory-tracking validation, NSGC is used to compute the generalized joint coordinates q individually. The addition of velocity/acceleration planning via interpolation or spline fitting would further improve the smoothness of the achieved trajectory. Furthermore, if the robot’s speed needs to be increased for high-dynamic use cases, using real-time trajectory generators instead of point-to-point stepping would also improve the timing control.

A drawback is the diverging radius as the robot configuration approaches a straight line (i.e., $\psi_{t}=\psi_{S}$ ). To avoid solving NSGC for extremely large radii (e.g., r>1,000 ), which exclusively occur for very slight bending (e.g., $|\psi_{t}-\psi_{S}|<$ 1°), the robot is considered to be in a straight configuration. Thus, end-effector orientation changes of less than 1° difference from $\psi_{S}$ cannot be realized with NSGC but are approximated as a straight configuration with $\psi_{t}=\psi_{S}$ .

Another limitation of this study is that only the positions were considered for trajectory tracking. In future studies, orientation should also be taken into consideration, as was done during the measurement of the individual target poses.

## Data Availability

Publicly available datasets were analyzed in this study. This data can be found here: https://github.com/SvenFritschResearch/NSGC/tree/main.
